# Severity-adjusted resource use and outcomes of an ICU of a tertiary hospital in Sao Paulo, Brazil

**DOI:** 10.1186/cc12631

**Published:** 2013-06-19

**Authors:** EGL Guadalupe, E Silva, F Colombari, A Serpa Neto, A Pardini

**Affiliations:** 1 Hospital Israelita Albert Einstein, Morumbi, São Paulo, SP, Brazil; 2 Hospital Alemão Oswaldo Cruz, Paraíso, São Paulo, SP, Brazil

## Introduction

Quality management in intensive care currently emphasizes outcome linked to optimization of resources. Intensive care medicine focuses on the most severe patients in the hospital and therefore high resource consumption, making its management a challenge. Of many parameters to estimate utilization of resources, only length of stay (LOS) is systematically collected. Therefore, severity-adjusted resource use (SRU) can be an important tool for estimating resource consumption in an ICU. The objective of this study was to evaluate the resource consumption by SRU in the adult ICU of the Hospital Israelita Albert Einstein, São Paulo, Brazil, in patients admitted during the first half of 2012.

## Methods

Retrospective analysis of 1,441 patients admitted during the first half of 2012 to a tertiary hospital in São Paulo, Brazil. Patients were divided into nine categories based on SAPS 3 as shown in Table 1, and then the standardized mortality rate (SMR), the quotient of observed to predicted mortality, was calculated. SRU was calculated for each stratum of SAPS 3 by dividing the LOS in the ICU for all patients by the number of survivors. The ICUs' SAPS 3 database was considered to standardize SRU. The amount of resources used was measured as the number of days per survival. Readmitted patients over 24 hours were excluded.

**Table 1:** SAPS 3 based on categories of severity

Category	1	2	3	4	5	6	7	8	9
SAPS 3 points	0 to 24	25 to 34	35 to 44	45 to 54	55 to 64	65 to 74	75 to 84	85 to 94	>95

## Results

A total of 1,441 patients were analyzed. The male proportion, age, LOS and SAPS 3 averages were 57.4%, 65 ± 18 years, 3.87 ± 5.95 days and 45.14 ± 15.9, respectively. The main results are shown in Table 2. No patient died of category 2.

**Table 2:** Data with SAPS 3, SRU and SMR

Category	SAPS 3 mean(SD)	Patients	Survivors	Σ days LOS (all patients)	SRU	SRU standardized	SMR
1	19.75 ± 4.08	112	111	161	1.45	0.03	1.1
2	30.36 ± 2.92	335	335	673	2.01	0.13	0
3	39.31 ± 2.77	350	346	823	2.37	0.16	0.13
4	49.18 ± 2.86	304	295	1,087	3.68	0.22	0.13
5	59.06 ± 2.79	202	174	1,155	6.64	0.23	0.3
6	68.77 ± 2.79	86	73	795	10.89	0.16	0.22
7	78.5 ± 2.45	34	18	251	13.94	0.05	0.56
8	88.54 ± 2.99	13	7	86	12.28	0.02	0.5
9	97.8 ± 4.21	5	2	16	8	0.003	0.62
Total	45.14 ± 15.9	1,441	1,361	5,047	3.72	0.13	0.24

## Conclusion

According to these data, the analyzed ICU appears to be very efficient for outcomes standardized by SAPS 3 and utilization of resources by SRU. However, confounding factors should be considered: decalibration SAPS 3 for this ICU and the presence of a step-down unit in the hospital.
